# Cross-Cultural Validation of the RECAP of Atopic Eczema Questionnaire in a Swedish Population

**DOI:** 10.2340/actadv.v104.38889

**Published:** 2024-06-19

**Authors:** Gunnthorunn SIGURDARDOTTIR, Mikael ALSTERHOLM, Chris D. ANDERSON, Maria BRADLEY, MariHelen SANDSTRÖM FALK, Emma K. JOHANSSON, Maria LUNDQVIST, Andreas SONESSON, Åke SVENSSON, Grigorios THEODOSIOU, Sophie VRANG, Laura B. von KOBYLETZKI

**Affiliations:** 1 Department of Dermatology and Venereology in Östergötland, and Department of Biomedical and Clinical Sciences, Linköping University, Linköping; 2 Department of Dermatology and Venereology, Institute of Clinical Sciences, Sahlgrenska Academy, University of Gothenburg, Gothenburg; 3 Division of Dermatology and Venereology, Department of Medicine Solna, Karolinska Institutet, Stockholm; 4 Dermatology, Karolinska University Hospital, Stockholm; 5 Vasakliniken Dermatology Clinic, Gothenburg; 6 Department of Dermatology and Venereology, Skåne University Hospital, Lund; 7 Division of Dermatology and Venereology, Department of Clinical Sciences, Lund University, Lund; 8 Department of Dermatology and Venereology, Skåne University Hospital, Malmö; 9 Patients’ organisation Atopikerna, The Swedish Asthma and Allergy Association, Stockholm; 10 Department of Occupational and Environmental Dermatology, Lund University, Skåne University Hospital, Malmö; 11 School of Medical Sciences, Faculty of Medicine and Health, Örebro University, Örebro, Sweden

**Keywords:** atopic dermatitis, eczema, patient-reported outcome measure, validation study

## Abstract

A Swedish translation of the patient-reported outcome measure for assessing long-term control of atopic dermatitis, Recap of atopic eczema (RECAP), has not been validated. Cross-cultural translation and multi-centre validation of the translated RECAP questionnaire were therefore performed. Disease severity was assessed using the validated Investigator Global Assessment Scale for atopic dermatitis (vIGA-AD^TM^). The Swedish RECAP was completed by 208 individuals aged 16 years or older with a median age of 36 years (interquartile range [IQR] 27–48). The participants considered the questionnaire suitable for assessing eczema control. The median RECAP score (range 0–28) was 12 (IQR 5–19). The mean and median vIGA-AD^TM^ scores (range 0–4) were 2 (standard deviation [SD] 2) and 3 (IQR 2–4), respectively. A correlation between RECAP and the vIGA-AD^TM^ was observed (*p* < 0.001). There was no significant change in scores for participants who answered the questionnaire twice within 14 days. Over time, improved or worsened eczema, as evaluated by vIGA-AD^TM^, affected RECAP scores significantly (*p* < 0.001). The study suggests that RECAP can assess AD control in a Swedish clinical setting and shows acceptable reliability.

SIGNIFICANCE

The Recap of atopic eczema questionnaire is a new long-term outcome measure to evaluate disease control in patients with atopic dermatitis. The Harmonizing Outcome Measures for Eczema initiative has recommended Recap of atopic eczema questionnaire as a core outcome measure to be used in all clinical trials. This validation enables the use of Recap of atopic eczema questionnaire to evaluate and compare long-term atopic dermatitis control in routine healthcare and clinical research in Sweden.

A topic eczema, also termed atopic dermatitis (AD), is a chronic inflammatory skin disease affecting children and adults. The 1-year prevalence is reported as 15–30% in children and 2–10% in adults (1–3). The severity of AD can vary significantly over time. Improved and sustained treatment effects of new systemic pharmacotherapies have created the need for a measure of long-term control of AD. Long-term control is recommended as a core outcome by the Harmonizing Outcome Measures for Eczema (HOME) initiative (4). A patient-reported outcome measure (PROM) capturing the patient’s long-term experience of AD control has been developed and validated in English (5). This 7-item instrument, Recap of atopic eczema (RECAP), has a score range of 0 to 28. Higher scores indicate poorer disease control. There are 2 versions of RECAP: a self-reported version for respondents from the age of 16 years and a caregiver-reported version for younger children.

A consensus process comparing available instruments to measure long-term AD control resulted in the recommendation by HOME for the use of the RECAP and Atopic Dermatitis Control Tool (ADCT) in clinical practice (6). Previous studies in the United Kingdom have found that RECAP has good validity and reliability (7, 8). However, the validity and reliability of questionnaires can vary across different populations and settings.

This study aimed to translate the original English RECAP questionnaire into Swedish and to conduct a cross-cultural validation of the Swedish version.

We hypothesised that the Swedish version of RECAP could be used in dermatology care settings and that self-reported AD control would correlate with disease severity assessed by the validated Investigator Global Assessment Scale for atopic dermatitis (vIGA-AD^TM^) (9).

## MATERIALS AND METHODS

### Study design

The study was a cross-sectional multicentre cross-cultural validation and feasibility study. It was conducted at 5 centres in Sweden: Skåne University Hospital in Malmö and Lund, Linköping University Hospital, Karolinska University Hospital in Stockholm, Sahlgrenska University Hospital and Capio Skin, Carlanderska Hospital in Gothenburg.

The study consisted of 2 parts: (*i*) cross-cultural questionnaire translation and (*ii*) questionnaire validation. Eligible inclusion criteria were AD diagnosis and age 16 years or older. An exclusion criterion was no written informed consent for participation in the study.

The study was approved by the Swedish Ethical Review Board (Dnr 2020-01341), and all participants gave written informed consent.

### Cross-cultural questionnaire translation

The RECAP questionnaire was translated from English to Swedish by 2 native Swedish speakers fluent in English. After that, these individuals agreed on a Swedish version of the questionnaire with AD patient representatives. The Swedish RECAP was then translated back into English by 2 native English speakers fluent in Swedish. The original and back-translated versions were compared, and all those involved in the translation process approved congruence with the original version. The translators were asked to assess whether the questionnaire was understandable, whether it captured the topic of eczema control, and to give suggestions for improvements. All authors approved the final version of the questionnaire.

### Validation of the questionnaire

The RECAP questionnaire was distributed consecutively to patients with AD aged 16 years or older at each participating centre. In addition to the questionnaire, the participants answered demographic questions regarding age, sex, education, and heredity for AD. Furthermore, the severity of AD was determined using the vIGA-AD^TM^. After answering the RECAP questionnaire, the participants evaluated the content validity of RECAP by filling out a separate semi-structured questionnaire. This questionnaire covered the participants’ views on the relevance of the content of RECAP. For each item, the participants assessed the difficulty of answering on a 4-point Likert scale (**Table I**). It was also possible to add comments. Lastly, the participants estimated how long it took to complete the RECAP questionnaire.

**Table I:** The participants’ assessment of the difficulty answering each item of the Recap of atopic eczema (RECAP) questionnaire (original English text is shown in the table instead of the Swedish version given to the participants)

Item	Not at all *n* (%)	A little difficult *n* (%)	Quite difficult *n* (%)	Very difficult *n* (%)
1. Over the last week, how has your eczema been?	141 (67.4)	46 (22.0)	20 (9.6)	2 (1.0)
2. Over the last week, on how many days has your skin been itchy because of your eczema?	144 (68.9)	36 (17.2)	20 (9.6)	9 (4.3)
3. Over the last week, on how many days has your skin been intensely itchy because of your eczema?	143 (68.4)	42 (20.1)	18 (8.6)	6 (2.9)
4. Over the last week, how much has your sleep been disturbed because of your eczema?	149 (71.3)	39 (18.7)	21 (10.0)	0 (0.0)
5. Over the last week, how much has your eczema been getting in the way of day-to-day activities?	144 (68.9)	50 (23.9)	14 (6.7)	1 (0.5)
6. Over the last week, on how many days has your eczema affected how you have been feeling?^a^	143 (68.7)	43 (20.7)	16 (7.7)	6 (2.9)
7. Over the last week, how acceptable has your eczema been to you?^a^	117 (56.2)	56 (26.9)	27 (13.0)	8 (3.9)

a One missing answer (*n* = 208).

**Fig. 1:**
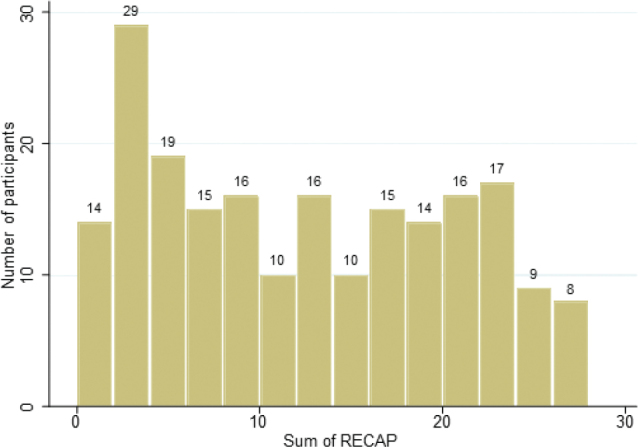
**RECAP scores.** Distribution of the RECAP scores shown as a number of participants for the sum of Recap of atopic eczema (RECAP), N 208.

Two smaller groups of participants answered the RECAP questionnaire twice to assess either test–retest reliability or the ability to change. The vIGA-AD^TM^ was used at all points as a comparator. For the test–retest reliability, the reply to the questionnaire should be the same or similar when answered twice within 14 days. For the ability to change, it is important that the outcome measure shows the ability to reflect changes in the score due to true differences when the symptoms change; therefore, an interval longer than 14 days between answering the questionnaire for the first and second time was chosen.

### Statistical analysis

A descriptive analysis of the data regarding RECAP’s content validity, feasibility, and reliability was performed. The χ^2^ test was used for all descriptive analyses, and *p*-values less than 0.05 were considered statistically significant. The correlation between vIGA-AD^TM^ and RECAP was tested with Spearman’s correlation coefficient. STATA 17 MP (StataCorp LLC, College Station, TX, USA) was used for statistical calculations.

## RESULTS

### Study population

The study included 209 individuals. The median age was 36 years (interquartile range [IQR] 27–48). Among the participants, 126 (60.3%) were women, and one identified as non-binary. More than half of the participants had a university degree (56.3%). A first-degree relative (parents and/or siblings) with AD was reported in 58.3%. The mean and median vIGA-AD^TM^ scores were 2 (standard deviation [SD] 2) and 3 (IQR 2–4), respectively, indicating mild to moderate disease.

### Participation

A total of 208 individuals completed RECAP and the accompanying semi-structured questionnaire addressing content validity. The RECAP results of 1 participant were excluded because the last question of the RECAP and the semi-structured questionnaire were not answered. Data for vIGA-AD^TM^ were missing for 1 participant.

### Content and face validity

The average time to complete the questionnaire was 3 minutes.

The RECAP questionnaire was deemed easy to understand by 202 (97.1%) of the participants. The items in the RECAP questionnaire were rated “not at all difficult to answer” by 56.3–71.3% of the participants (Table I). No item was considered challenging to answer, but the items concerning the number of days with itch and acceptability were deemed the most difficult. Even so, 144 (68.9%) and 117 (56.3%) of the participants reported that these items were not at all difficult to answer.

There were no specific queries regarding the layout of the questionnaire.

Written comments were left by a quarter (*n* = 49) of the participants. Many of the comments concerned the questions of acceptability of eczema and itch.

The comments reflected ambiguity regarding what could be perceived as acceptable on an individual level. One participant stated: “It is difficult to rate eczema when you have suffered from it your whole life. You have another starting point”. Another participant wrote: “It is difficult to assess eczema according to what should be reasonable and not according to what you have been used to”. Similar experiences were expressed regarding the assessment of itch: “… how many days the skin has been itching intensively was hard to answer because the difference between itch and intensive itch can be difficult to define”.

The participants seemed to experience a more significant challenge in assessing itch when it was mild to intermediate: “it was helpful to be able to relate [my itch] to a rating scale, such as ‘intensive itch’”.

There were also comments on the time of recollection. A few participants suggested extending the recall period: “Sometimes when it is time for an appointment with my doctor, the eczema has improved after a flare-up”. In contrast, participants also commented that it was difficult to recall an entire week because they “had not kept a diary”.

### RECAP scores

The median RECAP score was 12 (IQR 5–19). The participants used a range of response options. There was no evident floor or ceiling effect, with 1% of the participants having the lowest RECAP score and 1% having the highest RECAP score. The distribution of RECAP scores was close to normal distribution, with a slight shift towards lower scores.

### Reliability

To determine test–retest reliability, 18 (8.7%) participants answered the questionnaire twice within 14 days. There was no statistically significant difference between these RECAP scores. Over time, improved or worsened eczema affected RECAP scores significantly (*p* < 0.001).

There was a statistically significant association between RECAP and vIGA-AD^TM^ (*p <* 0.001). More severe disease (determined by vIGA-AD^TM^) was correlated with poorer AD control (determined by RECAP), Spearman’s *r_s_* 0.63 (*p <* 0.001).

## DISCUSSION

This study demonstrates that the Swedish version of RECAP has good acceptability, reliability, and validity in individuals with AD aged 16 years or older in specialist dermatological care. The questionnaire was considered easy to use and took only a few minutes to answer. This outcome of our study agrees with the results of the validation studies of the English version of RECAP. These studies have demonstrated a high completion rate, good content and convergent validity, and test–retest reliability in online surveys of over 300 adults and children recruited through social media, posters in public settings, and patient organizations (5,7). A high completion rate and convergent validity were also seen in another English study where participants were recruited at a hospital dermatology outpatient clinic. However, the sample size was smaller (27 adults and 16 children) (8).

In addition to English text, RECAP has previously been validated for Chinese, French (for Belgium), German, Dutch, Spanish, and Japanese translations (10). Content validity of the German and Spanish RECAP translations has been examined but only in qualitative studies. Like our results, German RECAP showed high comprehensibility, comprehensiveness, and relevance (11). The Spanish RECAP version was considered comprehensible by 15 adult patients who completed the Spanish RECAP and the ADCT (12). The 2 long-term eczema control instruments in the latter study showed strong correlation. Additionally, Gabes et al. found the self-reported English, German, and Dutch RECAP relevant and comprehensible in young people from 12 years of age (13).

The participants in the present study found the Swedish RECAP easy to understand and answer. Items regarding acceptability and frequency of itch were considered the most challenging to answer. A reason for this could be that many individuals with AD experience some degree of itch regularly or even continuously and come to regard this as an almost normal state.

In this study, the vIGA^TM^ was chosen as an anchor as the IGA is a validated “global” outcome measure for AD, reflecting the overall severity of AD. Further, the IGA has the benefit of being easily understood, accomplished, and useful in mild AD to severe AD. We did not ask the patients to answer other frequently used PROMs, such as the Patient Oriented Eczema Measure (POEM), which is a limitation. However, previous studies have shown a correlation between POEM and RECAP and between the Dermatology Life Quality Index (DLQI) and RECAP (5–7, 12). Furthermore, convergent validity between the vIGA-AD^TM^ and other PROMS, such as the POEM, has been previously demonstrated (14).

In our study, changes in RECAP scores were associated with changes in the physician-assessed severity score, vIGA-AD^TM^. In general, higher vIGA-AD^TM^ scores were related to higher RECAP scores. However, it did happen that participants assessed their disease control as decreased, although they had quite mild AD according to the vIGA-AD^TM^. This highlights the importance in clinical practice of discussing the results of rating scales with the patient and asking them to elaborate when discrepancies occur. Including another eczema severity measurement that takes into account the physician assessment but also the patient experience of itch, such as SCORAD, could have added to the understanding of the RECAP performance. One other study has correlated the scores of RECAP to physician-assessed disease severity scores including vIGA-AD^TM^ with similar results (15).

The strength of this study was that it included a high number of individuals from 16 years of age. The data sampling period was 13 months, covering seasonal variations. In contrast with previous validation studies of RECAP, where AD was self-reported, all patients were diagnosed and recruited at a dermatology department, reinforcing the certainty of the diagnosis.

In summary, the Swedish version of RECAP for AD has good validity, reliability, and responsiveness to change in respondents from 16 years of age. Perceived difficulty in rating itch or defining what is acceptable might be explained by the challenge of assessing long-term effects of chronic disease rather than poor comprehensibility. Nevertheless, an additional rating scale for itch could have been more explicit.

Large-scale cross-cultural validation studies of questionnaires in languages other than English for both children and adults are essential for standardised evaluation of disease control and to enable comparisons of treatment effects in different countries and cultures. The Swedish version of RECAP will facilitate future evaluation of long-term disease control in AD patients.
